# Evidence of Better Autonomic, Metabolic and Psychological Profile in Breast Cancer Survivors Meeting Current Physical Activity Recommendations: An Observational Study

**DOI:** 10.3390/jpm12020273

**Published:** 2022-02-13

**Authors:** Daniela Lucini, Mara Malacarne, Wolfgang Gatzemeier, Eleonora Pagani, Giuseppina Bernardelli, Gianfranco Parati, Massimo Pagani

**Affiliations:** 1 BIOMETRA Department, University of Milan, 20129 Milan, Italy; mara.malacarne@unimi.it; 2 Exercise Medicine Unit, Istituto Auxologico Italiano, IRCCS, 20135 Milan, Italy; massimo.paganiz@gmail.com; 3 Cancer Center, Humanitas Clinical and Research Center, 20089 Rozzano, Italy; wolfgang.gatzemeier@humanitas.it; 4 Department of Psychology, Catholic University of the Sacred Hearth, 20123 Milan, Italy; eleonora.pagani@unicatt.it; 5 DISCCO Department, University of Milan, 20122 Milan, Italy; g.bernardelli@unimi.it; 6 Department of Medicine and Surgery, University of Milano-Bicocca, 20126 Milan, Italy; gianfranco.parati@unimib.it; 7 Department of Cardiology, Istituto Auxologico Italiano, IRCCS, 20145 Milan, Italy

**Keywords:** autonomic nervous system, prevention, exercise, nutrition, heart rate variability

## Abstract

The increased cardiometabolic risk observed in breast cancer survivors (BCS) is due to multiple mechanisms: Hormonal and immunological dysfunction are well-identified ones, while cardiac autonomic regulation (CAR) is less recognized but may play a new complementary role particularly relevant when considering conditions and behaviors associated with a better prognosis in BCS, such as physical training. This observational study investigated a group of consecutive (172) BCS subdivided in two groups: those who reached the physical activity goals above 600 (MET·min/week) and those who did not. We assessed CAR by autoregressive spectral analysis of cardiovascular variabilities (considering in particular the unitary autonomic nervous system index—ANSI), body mass composition, stress perception and lifestyle in order to verify possible differences due to execution of physical activity. Subjects who spontaneously met physical activity recommendations presented a better autonomic, metabolic and psychological profile compared to those who did not. Lower physical activity volume, poor metabolic parameters, increased stress and fatigue perception may cluster together, leading to worsened CAR. This control mechanism may play a complementary role in determining the increased cardiometabolic risk observed in BCS. Furthermore, it may also explain, albeit in part, the better prognosis observed in patients following interventions aiming to improve the sympathetic–parasympathetic balance, such as physical training, using a personalized medicine approach.

## 1. Introduction

Breast cancer survival has improved during recent decades [1] as a result of early diagnosis and advances in cancer treatment. Paradoxically, this progress is at risk of being offset by the potential late-occurring cardiovascular toxicity of oncologic treatment and worsening of cardiometabolic profile [2], which might favor over time the occurrence of chronic conditions jeopardizing survivors’ wellbeing.

Many factors may contribute to the worsening of cardiometabolic profile, ranging from specific side effects of adjuvant therapies to the patient’s exercise/nutrition habits and psychological profile [2]. Lack of exercise, poor nutrition, overweight and obesity, smoking and stress are well known elements which increase the risk of overall mortality and reduce quality of life, both in the general population [3] and in breast cancer patients [2]. The mechanisms responsible for this increased risk are multiple [2], some of which are well identified, such as hormonal and immunological dysfunction; others, such as alteration in cardiac autonomic regulation (CAR), are less recognized but may play a new complementary role [4,5,6,7,8,9]. Moreover, alterations of CAR (modeled according to a competing sympathetic and parasympathetic dynamical interaction) may per se characterize several cardiometabolic diseases such as hypertension, coronary artery disease, obesity and diabetes [10,11,12,13]. Furthermore, data are presented in literature demonstrating a role of CAR dysfunction even in cancer [4,5,8,9,14]. It is important to consider that aerobic exercise training, weight and stress reductions and stopping smoking [15,16,17,18] may improve CAR with consequent reduction in cardiometabolic risk and prognosis. In particular, aerobic exercise training is associated, in cardiac/diabetic/obese patients and healthy subjects, with improved vagal and reduced sympathetic control of cardiac function [16,17,18,19], and progressive levels of cardiorespiratory fitness (CRF) are strongly associated with proxies of improved CAR [20]. These findings are important considering that a growing body of clinical evidence shows that low levels of CRF are associated with a high risk of cardiometabolic disease, all-cause mortality and mortality rates attributable to various cancers and that reduced CRF is a potentially stronger predictor of mortality than established risk factors [21,22,23]. Vice versa, high levels of CRF are associated with a reduction in cardiometabolic diseases and even cancer [23,24]. Multiple studies addressed the relationship between exercise and cancer, demonstrating that to be physically active is of pivotal importance in the primary and secondary prevention of cancer [25,26]. Considering secondary prevention in breast cancer survivors, exercise is associated with significant decrease in risks of total and cancer-related mortality, both before and after cancer diagnosis [6,25,27].

Few data are present in literature regarding the CAR in breast cancer survivors considering the level of physical activity performed. In this observational study we hypothesized that breast cancer survivors who are physically active, meeting current physical activity recommendations, are characterized by a better cardiac autonomic regulation, and that this can be easily assessed in a clinical setting using a novel unitary autonomic nervous system index (ANSI) [13,28,29,30] which is presented as % rank. This index has been shown [13] to be by design insensitive to age and gender, hence overcoming some major problems of interpretation of data derived from non-invasive assessment of CAR using spectral analysis of heart rate variability.

The goal of this study was to verify if physically active breast cancer survivors are characterized by a better cardiac autonomic regulation as compared with sedentary ones.

## 2. Materials and Methods

**Figure 1:**
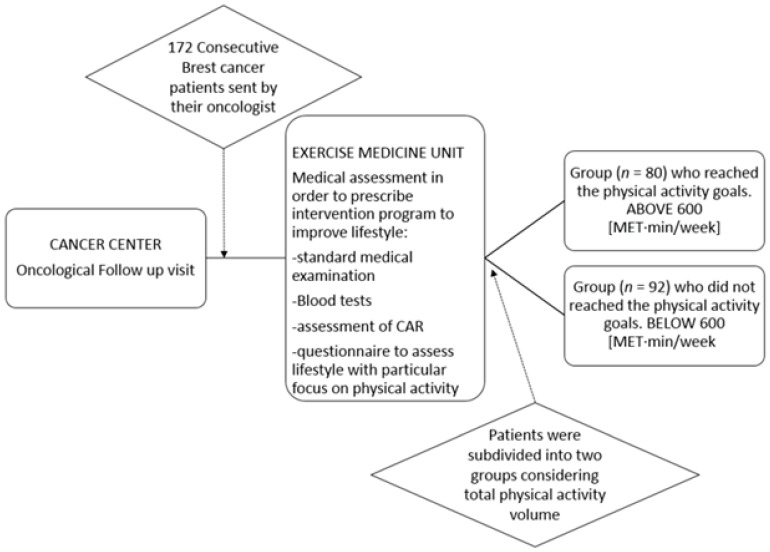
Flow chart: sample selection and protocol.

Design, setting and participants:

In this observational, proof-of-concept study we considered a group of consecutive breast cancer survivors (*n* = 172) who subsequently attended the Exercise Medicine Unit (as suggested by their oncologist) in order to start a modification program aimed at improving lifestyle and hence reducing cardiometabolic–oncologic risk (see Figure 1).

Patients were treated at the Humanitas Research Hospital Cancer Center following best clinical practice. Eligibility criteria included age (30–75 years), no evidence of metastases (as per routine personalized follow up program considering radiological examinations) and absence of acute conditions (within the past three months). General average characteristics are reported in Table 1. None of the patients underwent CT or RT treatments during the study period; they proceeded with endocrine therapy and/or biological (trastuzumab) therapies. Endocrine therapy (inducing estrogen inhibition) was considered the selective estrogen receptor modulator (SERM) tamoxifen, LH-RH analogue or aromatase inhibitors. The choice of the patient’s treatment protocol was based on patients’ and/or tumor characteristics.

Ethical considerations:

Informed consent was obtained from all individuals participating in the study. The protocol of this study followed the principles of the Declaration of Helsinki and Title 45, US Code of Federal Regulations, Part 46, Protection of Human Subjects, Revised 13 November 2001, effective 13 December 2001, and was approved by the local Institutional Ethics Committee (letter signed by Humanitas Independent Ethics Committee 13 October 2015). All participants at the time of first clinical assessment signed an agreement to use their anonymized data for population studies and possible publications. They acknowledged that they cannot be identified via the paper and that authors had fully anonymized their data.

All subjects underwent the following assessments.

### 2.1. Clinical Assessment

- History, standard medical examination, anthropometric and hemodynamic data;
- Blood tests (see Table 1);
- BIA (Bioelectrical Impedance Analysis; BodyStat Quadscan 4000, BodystatR Quadscan 4000, Body Stat Ltd., Isle of Man, British Isles) was employed in order to estimate percentage of fat mass (FM) and of free fat mass (FFM) using the proprietary equation provided by the manufacturer [31].

### 2.2. Cardiac Autonomic Regulation (CAR)

Our approach to the non-invasive evaluation of autonomic regulation has recently been summarized [32]. In brief, ECGs, non-invasive (Finometer, TNO, The Netherlands) arterial pressure and respiratory activity (piezoelectric belt, Marazza, Italy) were acquired on a PC. Beat-by-beat data series obtained during 5 min rest and subsequently during 5 min standing were analyzed offline with dedicated software that provides time and frequency domain indices of heart rate variability.

Recently, to simplify clinical interpretation of multiple heart rate variability (HRV) indices [33], we described a unitary autonomic index (ANSI) [13]. Computation of ANSI depends on the combination of principal factor analysis and clinically optimized radar plot, considering the cardiac autonomic information carried by RR, RR interval variance and changes in low frequency heart rate spectral powers, expressed in normalized units (ΔLFnu) [13]. The computing procedure first corrects for age by percentile rank transformation, second ranks the information (82.7% of variance accounted for) distributed across indices from the selected clusters of variability (considering amplitude and oscillatory code modalities [32]) and third using a radar plot [13] builds ANSI as a composite [33] triangle area that is finally percent ranked against the benchmark population. ANSI is treated as a proxy of cardiac autonomic regulation.

### 2.3. Lifestyle Assessment

#### 2.3.1. Physical Activity

Physical activity (weekly physical activity volume) was assessed by a modified version of the commonly employed short version of the International Physical Activity Questionnaire [34,35], which focuses on intensity (nominally estimated in metabolic equivalents—METs—according to the type of activity) and duration (in minutes) of physical activity. We considered the following levels: brisk walking (≈3.3 METs), other activities of moderate intensity (≈4.0 METs) and activities of vigorous intensity (≈8.0 METs). In accordance with current guidelines [36], these levels were used to assess adherence to guideline weekly exercise volume, using the following equations:

- Moderate intensity (MET·min/week) = (3.3 × min of brisk walking × days of brisk walking) + (4.0 × min of other moderate intensity activity × days of other moderate intensity activities);
- Vigorous intensity: (MET·min/week) = 8.0 × min of vigorous intensity activity × days of vigorous intensity activity;
- Total weekly physical activity volume (MET·min/week) = sum of moderate + vigorous MET·min/week scores.

The population of our study was subdivided into two groups: those (*n* = 80) reaching the physical activity goals as suggested by the latest guidelines [36,37] corresponding to 150 min/week of moderate activity, 75 min/week of vigorous activity or a combination of both (above 600 (MET·min/week) considering total weekly physical activity volume), and those (*n* = 92) who did not reach the physical activity goals (Below 600 (MET·min/week) considering total weekly physical activity volume).

#### 2.3.2. Nutrition

Nutrition was assessed using the AHA Diet Score [38], taking into consideration fruit/vegetables, fish, sweetened beverages, whole grain and sodium consumption (the assessment of which was adapted to Italian eating habits).

#### 2.3.3. Stress Perception

Perception of stress, fatigue, control and somatic symptoms (4SQ) was assessed using a self-administered questionnaire [39,40,41] providing nominal self-rated Likert scales from 0 (no perception) to 10 (highest perception) for each measure. The 4SQ questionnaire considers 18 somatic symptoms; thus, the total score ranged from 0 to 180.

### 2.4. Statistical Analysis

Data are presented as mean ± standard deviation (SD). Differences between the two considered groups were assessed with unpaired t-test or chi-square. Spearman’s simple correlations and automatic linear modeling were also employed. Computations were performed with a commercial package (IBM SPSS 26) considering *p* < 0.05 as the significance threshold.

## 3. Results

**Figure 2:**
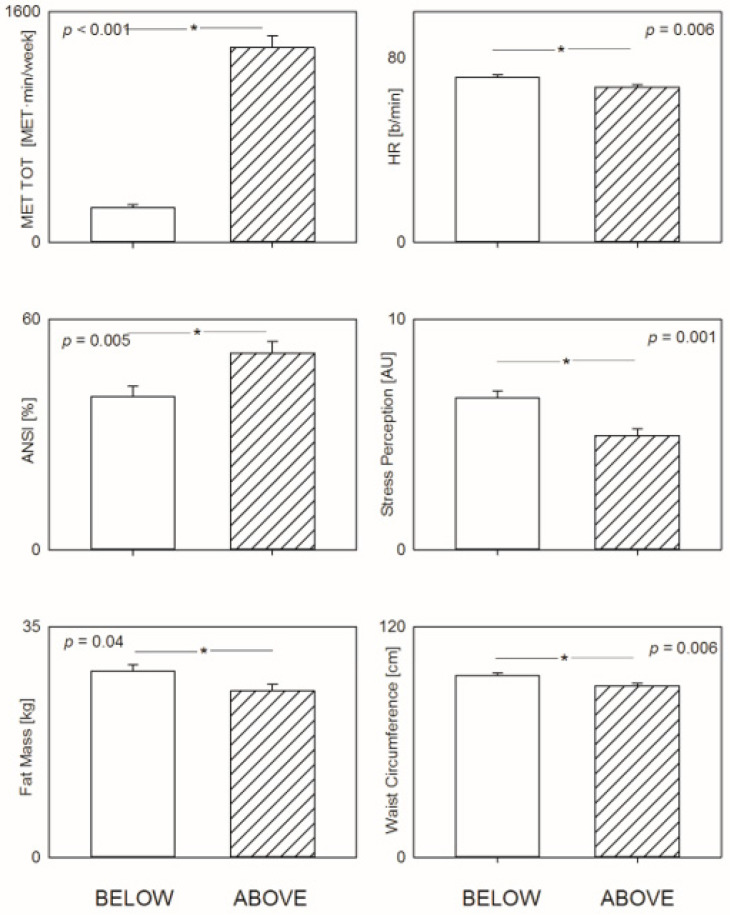
Differences in the main selected parameters between breast cancer survivors who spontaneously met (ABOVE) current physical activity goals and those who did not (BELOW). Physically active patients showed a better autonomic, metabolic and psychological profile. ET TOT = total weekly activity volume, * = *p* significance.

The percentages of patient who underwent chemotherapy/radiotherapy or were treated with endocrine and/or immunological therapies were similar in the two considered groups; likewise, the percentages of patients with dyslipidemia, hypertension or hypothyroidism were similar (Table 1). Metabolic parameters (weight (*p* = 0.003), body mass index (*p* = 0.005), waist circumference (*p* = 0.006), fat mass (kg) (*p* = 0.040) and total body water (kg) (*p* = 0.002),) were lower in the group of patients who spontaneously met current physical activity goals. This group presented also lower scores of stress (*p* = 0.001), fatigue (*p* = 0.012) and somatic stress-related symptoms (4SQ) (*p* = 0.026). No significant differences were observed in AHA Diet Score, perception of control or lipid or fasting plasma glucose levels (Table 1 and Figure 2).

Heart rate (*p* = 0.015) and the marker of prevalent sympathetic modulation of the SA node (RR LFnu) (*p* = 0.021) were lower in patients who met current physical activity goals, while the marker of prevalent parasympathetic modulation to the SA node (RR HFnu) (*p* = 0.015) and the unitary autonomic nervous system index (ANSI) (*p* = 0.005) were higher (see Table 2 and Figure 1).

**Figure 3:**
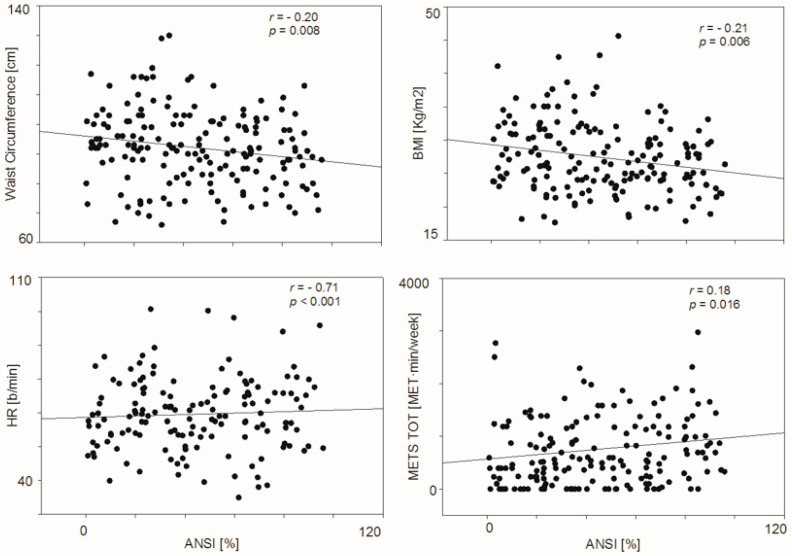
Scatterplot ANSI against waist circumference, BMI, heart rate and total activity volume (METs TOT).

Table 3 reports a simple correlation matrix of ANSI, anthropometric, metabolic, activity and stress indices. In spite of the small population, it is clear that weekly physical activity volumes (particularly the total activity volume) were significantly correlated with ANSI (*p* = 0.016), heart rate (*p* = 0.033), metabolic parameters, scores of stress and diet (*p* = 0.010). Moreover, ANSI was significantly correlated with anthropometric and metabolic parameters (see Table 3 and Figure 3).

## 4. Discussion

In this observational study we showed that breast cancer survivors who spontaneously meet current physical activity recommendations present a better autonomic, metabolic and psychological profile as compared to breast cancer survivors who do not.

Exercise training represents a pivotal strategy to reduce cardiometabolic and cancer risk both in primary and secondary prevention [23,24,25,26]. Moreover, in breast cancer survivors, it represents a convenient tool to contrast some important side effects of adjuvant therapy such as increased body weight and increased fatigue [42,43,44]. In our study we observed that patients who were physically active were characterized by lower body weight, lower BMI, reduced fat mass and lower waist circumference, resulting in a better metabolic profile, while no differences were noted regarding adjuvant therapy regimen. Of particular interest is the observation of an improved cardiac autonomic regulation as indicated by the greater ANSI, higher marker of prevalent vagal modulation to the sinoatrial node (RR HFnu), lower heart rate and lower marker of prevalent sympathetic modulation of the sinoatrial node (RR LFnu) as compared to patients who are not physically active. In other groups of subjects, such as cardiac or metabolic patients, a better CAR, as consequence of aerobic training, is associated with a more favorable prognosis and with a lower cardiometabolic and overall mortality [15,16,17,18,19]. The mechanisms responsible for this improvement are multifarious. Exercise may directly influence autonomic nervous system control and may act indirectly reducing metabolic risk factor such as overweight and fat mass. Indeed, a lower fat mass may per se contribute to a betterment of CAR as observed in subjects losing fat mass after nutrition programs [19]. This may be important clinically because the interaction of several different favorable mechanisms may result in unexpected greater reductions in risk, producing a “risk factor gap” [15].

Regarding patients of this study specifically, automatic linear modeling suggests that BMI, fat mass, stress somatic symptoms and physical activity (as measured by moderate and intense loads) individually predict CAR (as represented by the proxy ANSI) with a progressively lower importance (from 0.49 to 0.10).

Accordingly, this observation might help to better delineate the use of ANSI as a proxy of CAR in a clinical setting. On one hand, from the neurovegetative side, we must recall that ANSI is built as a unitary indicator combining the sympatho-vagal information carried by HRV and distributed in three principal domains coding for different aspects of the variability phenotype (pulse, oscillations and amplitude) [32]. From Table 2 it appears that, in the present population, pulse and oscillations are significantly different according to physical activity volumes, while amplitude (here represented by RR variance) is not significantly modified. A lower discriminant capacity of RR variance as compared to normalized oscillatory components of beat-by-beat variability of RR interval has already been reported [45]. Moreover, nonlinear measures (see Table 2) such as the normalized index of regularity (Ro) based on an entropy rate (i.e., the conditional entropy) and the deterministic patterns (RRP_0v, RRP_1v, RRP_2lv, RRP_2uv) lasting three beats in four categories according to the number and type of heart period changes are not significantly different. In addition, and importantly, the index ANSI is by design insensitive to age and gender and being presented as % rank (range 0–100) is (conveniently) of immediate interpretation; in normal conditions higher values indicate a better performance of the sympathetic–parasympathetic balance [13].

In this study activity volume was assessed by a modified version of the commonly employed short version of the International Physical Activity Questionnaire [35,46]; we do not have any direct measure of performed exercise. We recently reported that normal subjects meeting the recommended weekly activity goals have higher fitness indicators (such as VO2max) combined with evidence of an autonomic shift towards parasympathetic prevalence [20]. Breast cancer survivors are generally characterized by a reduced cardiorespiratory fitness [23,24], and its improvement with an active lifestyle may be responsible for the better autonomic profile contributing to a reduced cardiovascular risk. Another important issue to consider is the important link between stress, fatigue and physical activity. We observed in the subjects whose nominal weekly physical activity volume is above the suggested value of 600 METs/week a significantly reduced perception of stress and fatigue, with no differences in adjuvant therapy regimen (see Table 2). Moreover, significant correlations (see Table 3) were found between total activity volume, stress and fatigue perception. The presence of stressful events per se may play a further role in the increased cardiovascular risk [47] both acting indirectly by worsening lifestyle and directly by impairing body control systems such as the immunological, hormonal and autonomic ones [48]. Of particular interest is the reduced CAR characterizing stressful situations [40,48]. On the other hand, the capability to maintain/assume a healthy lifestyle (being physically active) in stressful conditions, such as breast cancer, may represent a tool to manage stress perception and the negative stress effects on control systems, such as autonomic control, representing a real tool to improve quality of life and prognosis [49].

A healthy lifestyle may also be a “countermeasure” in those patients who need to assume some adjuvant therapies which may affect negatively CAR. In a previous paper [30] we observed in breast cancer survivors that endocrine adjuvant therapy was associated with a more apparent impairment in autonomic regulation, considering a group of breast cancer survivors who were, at the time of evaluation, undergoing long term adjuvant endocrine therapy (11.7% were also treated with trastuzumab) and breast cancer survivors who were not on long term endocrine therapy at the time of the evaluation (11.8% were also treated with trastuzumab). In the present study the percentages of patients treated with tamoxifen, LH-RH analogue, aromatase inhibitors or trastuzumab were similar, suggesting that the difference in CAR observed may not be considered a main consequence of different treatment protocol but a plausible consequence of a different volume of physical activity performed. Unfortunately, the number of patients treated with different drugs (also considering that some patients were undergoing both endocrine and trastuzumab therapies and that some patients were undergoing different endocrine treatments) was too small to perform statistical analysis in order to detect possible differences in CAR.

The possibility to easily and conveniently demonstrate cardiac autonomic regulation may represent a strength of this paper; of particular interest may be the use of this methodology in a clinical setting with patients, such as breast cancer survivors, who may benefit from interventions based on exercise training, which may improve CAR.

On the other hand this study presents some weaknesses/limitations: It was an observational study; we did not obtain any objective measure of physical activity; rather, it was only estimated using validated questionnaires; we did not obtain parameters on immunological and hormonal controls which play an important role in the etiopathogenesis of cancer and which may be ameliorated by exercise training and the size of the sample population was too low to permit statistical analysis to study a possible different effect of exercise CAR in patients treated with different drug regimens.

## 5. Conclusions

In conclusion, the results of our study show that breast cancer survivors who spontaneously meet current physical activity recommendations are characterized by a better autonomic, metabolic and psychological profile as compared to breast cancer survivors who do not. Lower physical activity volume, poor metabolic parameters, increased stress and fatigue perception may cluster together, leading to a worsening of CAR. These mechanisms may play a complementary role [50] in determining the increased cardiometabolic risk observed in breast cancer survivors and might provide an explanation for the “risk factor gap” [15], supporting interventions aiming to improve the sympathetic–parasympathetic balance such as lifestyle management. ANSI might thus offer a simple metric to quantify CAR in a clinical setting, using a personalized medicine approach.

## Figures and Tables

**Table 1:** Anthropometric, hemodynamic, metabolic and behavioural data observed in patients whose weekly physical activity was below (*n* = 92) or above (*n* = 80) the current physical activity goals.

Variable	Below			Above			*p*
Age	49.77	±	9.06	50.41	±	8.28	0.63
Smoking (*n*; %)	10; 10.9			11; 13.8			0.56
Time since surgery (months)	26.5	±	27.4	29.8	±	32.7	0.47
RT (*n*; %)	69; 75			60; 75			0.57
CT (*n*; %)	62; 67.4			46; 57.5			0.18
Current endocrine therapy (tamoxifen) (*n*; %)	28; 30.4			27; 33.7			0.64
Current endocrine therapy (LH-RH analogue) (*n*; %)	25; 27.2			18; 22.5			0.48
Current endocrine therapy (aromatase inhibitors) (*n*; %)	32; 34.8			26; 32.5			0.75
Current trastuzumab (*n*; %)	12; 13			8; 10			0.53
Hypertension (*n*; %)	10; 10.8			8; 10.0			0.85
Hypothyroidism (*n*; %)	3; 3.3			5; 6.2			0.47
Dyslipidemia (*n*; %)	7; 7.6			6; 7.5			0.97
SAP (mmHg)	117.32	±	15.39	118.99	±	15.65	0.48
DAP (mmHg)	78.04	±	9.76	76.81	±	9.58	0.40
HR (b/min)	71.80	±	10.37	67.94	±	10.19	0.015
Activity volume (moderate) (MET·min/week)	241.01	±	206.30	1237.41	±	578.70	<0.001
Activity volume (vigorous)(MET·min/week)	0.00	±	0.00	115.00	±	631.83	0.10
Total activity volume (moderate + vigorous) (MET·min/week)	241.01	±	206.30	1352.41	±	744.39	<0.001
AHA Diet Score (a.u.)	2.22	±	0.94	2.46	±	0.91	0.08
Weight (kg)	73.87	±	13.87	67.68	±	12.43	0.003
Height (cm)	1.61	±	0.06	1.61	±	0.08	0.69
BMI (kg/m^2^)	28.45	±	5.41	26.22	±	4.79	0.005
Waist circumference (cm)	94.54	±	12.82	89.32	±	11.88	0.006
Fat mass (kg)	28.28	±	9.54	25.29	±	8.53	0.040
Free fat mass (kg)	44.52	±	6.42	42.74	±	5.53	0.06
Total body water (kg)	34.44	±	4.03	32.64	±	3.01	0.002
Fat mass (%)	37.78	±	6.87	36.43	±	7.02	0.22
Free fat mass (%)	62.34	±	6.97	63.61	±	7.01	0.25
Total body water (%)	47.52	±	4.93	48.78	±	5.54	0.13
Total cholesterol (mg/dL)	214.7	±	41.95	215.02	±	42.21	0.48
HDL cholesterol (mg/dL)	61.37	±	16.11	62.43	±	15.31	0.46
LDL cholesterol (mg/dL)	129.61	±	33.72	129.94	±	39.86	0.13
Triglycerides (mg/dL)	101.42	±	54.08	102.26	±	44.60	0.71
Fasting glucose (mg/dL)	92.08	±	12.89	91.98	±	9.40	0.055
Stress perception (au)	6.59	±	2.97	4.96	±	3.35	0.001
Fatigue perception (au)	6.72	±	2.77	5.58	±	3.15	0.012
Control perception (au)	6.52	±	2.27	6.45	±	2.70	0.85
4SQ Score (au)	51.43	±	28.86	41.56	±	28.71	0.026

Data are presented as mean ± SD with *p* significance according to unpaired *t*-test or chi-square. Abbreviations: *p* = significance; RT = radiotherapy; CT = chemotherapy; BMI = body mass index; SAP = systolic arterial pressure; DAP = diastolic arterial pressure; HR = heart rate; AHA = American Heart Association; MET = metabolic equivalent; HDL = high density lipoprotein; LDL = low density lipoprotein; 4SQ = Subjective Somatic Stress Symptoms Questionnaire.

**Table 2:** Values of cardiac autonomic proxies observed in patients whose weekly physical activity was below or above the current physical activity goals.

Variable	Below			Above			*p*
RR (ms)	855.57	±	123.13	912.06	±	143.88	0.006
RR VAR (ms^2^)	1440.08	±	1874.00	1591.89	±	1767.47	0.58
RR LFa (ms^2^)	379.66	±	675.74	355.39	±	500.39	0.78
RR HFa (ms^2^)	359.21	±	836.57	447.61	±	714.28	0.45
RR LFnu (nu)	52.93	±	22.87	44.89	±	22.08	0.021
RR HFnu (nu)	40.14	±	20.94	48.14	±	21.57	0.015
RR LF/HF	4.64	±	16.80	2.49	±	7.05	0.26
RRRo (au)	0.33	±	0.11	0.31	±	0.10	0.38
RRP_0v (%)	28.85	±	14.73	25.59	±	14.61	0.14
RRP_1v (%)	46.50	±	6.74	47.69	±	7.58	0.28
RRP_2lv (%)	8.90	±	6.07	9.51	±	6.87	0.54
RRP_2uv (%)	15.75	±	10.53	17.21	±	10.18	0.35
A.XXAR (ms/mmHg)	3.36	±	2.95	3.49	±	3.63	0.81
SAP Mean (mmHg)	120.41	±	18.11	122.84	±	19.44	0.40
SAP LFa (mmHg^2^)	4.30	±	5.75	5.22	±	14.85	0.60
α Index (ms/mmHg)	13.53	±	9.50	13.93	±	10.15	0.78
ANSI (%)	39.84	±	25.49	51.28	±	27.37	0.005

Abbreviations: *p* = significance; RR = average of RR interval from tachogram sections; RR VAR = variance from tachogram sections; LFa = absolute power (a) of low frequency (LF) component of RR variability; RR HFa = absolute power (a) of high frequency (HF) component of RR variability; RR LFnu = normalized power (nu) of low frequency (LF) component of RR variability; RR HFnu = normalized power (nu) of high frequency (HF) component of RR variability; RR LF/HF = ratio between absolute values of LF and HF; Ro = regularity index; p_0v, p_1v, p_2l, p_2v = deterministic patterns; A.XXAR = index of arterial baroreflex; SAP mean = systolic arterial pressure by Finometer; α Index = index of overall cardiac baroreflex sensitivity; ANSI = composite index of cardiac autonomic regulation.

**Table 3:** Spearman’s correlation within selected variables.

ANSI	1.000												
HR	−0.712 **	1.000											
	**0.000**												
FM (kg)	−0.104	0.084	1.000										
	0.190	0.292											
WEIGHT	−0.162 *	0.142	0.897 **	1.000									
	**0.034**	0.064	**0.000**										
BMI	−0.208 **	0.156 *	0.929 **	0.881 **	1.000								
	**0.006**	**0.041**	**0.000**	**0.000**									
WC	−0.201 **	0.145	0.872 **	0.831 **	0.863 **	1.000							
	**0.008**	0.057	**0.000**	**0.000**	**0.000**								
METs M	0.173 *	−0.147	−0.143	−0.199 **	−0.198 **	−0.175 *	1.000						
	**0.023**	0.054	0.072	**0.009**	**0.009**	**0.022**							
METs V	0.178 *	−0.168 *	−0.077	−0.150 *	−0.121	−0.136	0.039	1.000					
	**0.020**	**0.027**	0.334	**0.050**	0.114	0.076	0.608						
METs TOT	0.183 *	−0.163 *	−0.166 *	−0.232 **	−0.210 **	−0.197 **	0.964 **	0.226 **	1.000				
	**0.016**	**0.033**	**0.036**	**0.002**	**0.006**	**0.010**	**0.000**	**0.003**					
STRESS	−0.086	0.013	0.029	−0.007	0.015	−0.029	−0.265 **	−0.070	−0.242 **	1.000			
	0.261	0.862	0.719	0.923	0.846	0.707	**0.000**	0.364	**0.001**				
FATIGUE	−0.147	0.103	0.067	0.036	0.026	−0.011	−0.180 *	−0.042	−0.158 *	0.692 **	1.000		
	0.054	0.180	0.404	0.644	0.737	0.890	**0.018**	0.585	**0.038**	**0.000**			
4SQ	−0.125	0.113	−0.002	−0.036	−0.017	−0.044	−0.146	−0.118	−0.141	0.548 **	0.578 **	1.000	
	0.102	0.141	0.979	0.643	0.827	0.570	0.055	0.123	0.064	**0.000**	**0.000**		
AHA Score	0.149	−0.089	−0.030	−0.040	−0.060	−0.009	0.177 *	0.067	0.195 *	−0.079	−0.078	−0.089	1.000
	0.051	0.248	0.704	0.607	0.432	0.908	**0.020**	0.385	**0.010**	0.301	0.310	0.243	
	**ANSI**	**HR**	**FM (kg)**	**WEIGHT**	**BMI**	**WC**	**METs M**	**METs V**	**METs TOT**	**STRESS**	**FATIGUE**	**4SQ**	**AHA Score**

Bold values indicate significant correlations. * Correlation is significant at the 0.05 level (two-tailed). ** Correlation is significant at the 0.01 level (two-tailed). Abbreviations: ANSI = autonomic nervous system index; HR = heart rate; FM = fat mass; BMI = body mass index; WC = waist circumference; MET = metabolic equivalent; M = moderate; V = vigorous; TOT = total activity volume; 4SQ = Subjective Somatic Stress Symptoms Questionnaire; AHA Score = American Heart Association Diet Score.

## Data Availability

Data are available upon request to the corresponding author.
